# ‘Opportunity to bond and a sense of normality’: Parent and staff views of cuddling babies undergoing therapeutic hypothermia in neonatal intensive care: ‘CoolCuddle’

**DOI:** 10.1111/hex.13477

**Published:** 2022-03-24

**Authors:** Jenny Ingram, Lucy Beasant, David Odd, Ela Chakkarapani

**Affiliations:** ^1^ Centre for Academic Child Health, Population Health Sciences University of Bristol Bristol UK; ^2^ Population Medicine Cardiff University School of Medicine Wales UK; ^3^ Neonatology, St Michael's Hospital University Hospitals Bristol and Weston NHS Foundation Trust Bristol UK

**Keywords:** neonatal intensive care units, parent–infant bonding, qualitative interviews, therapeutic hypothermia

## Abstract

**Background:**

Currently, parents whose sick babies are undergoing three days of cooling therapy for hypoxic–ischaemic encephalopathy in neonatal intensive care units (NICUs) are not permitted to cuddle their cooled babies, due to concerns of warming the baby or dislodging breathing tubes or vascular catheters. Parents want to stay and care for their cooled babies and have reported that bonding is adversely affected when they are not permitted to hold them.

**Design and Participants:**

Qualitative interviews with 21 parents of cooled babies in NICU (11 mothers and 10 fathers) and 10 neonatal staff (4 consultants and 6 nurses) explored their views and experiences of an intervention to enable parents to cuddle their cooled babies (CoolCuddle). Thematic analysis methods were used to develop the themes and compare them between parents and staff.

**Results:**

Five themes were produced. Three themes were comparable between parents and staff: Closeness, a sense of normality and reassurance and support. An additional parent theme reflected their mixed feelings about initial participation as they were apprehensive, but felt that it was an amazing opportunity. Parents and staff described the closeness between parents and babies as important for bonding and breastfeeding. Fathers particularly appreciated the opportunity to hold and bond with their infants. Parents valued the reassurance and support received from staff, and the cuddles helped them feel more normal and more like a family at a very stressful time. In a final staff theme, they discussed the skills, number of staff and training needed to undertake CoolCuddle in NICU.

**Conclusions:**

Parents cuddling their babies during cooling therapy enhanced parent–infant bonding and family‐centred care in NICU and was positively received. Adverse perinatal mental health, impaired mother–infant bonding and their effects on the establishment of breastfeeding may be ameliorated by introducing CoolCuddle.

**Patient Contribution:**

Our parent advisors contributed to the interview topic guides and endorsed the themes from the analysis.

## 1 BACKGROUND

In the United Kingdom, each year, over 2000 babies experience brain injury at birth, due to lack of oxygen and blood supply (hypoxic–ischaemic encephalopathy [HIE] and receive 72 h of cooling treatment and intensive care to reduce the risk of death or disabilities.^1^, ^2^ These babies typically require resuscitation and stabilization after birth and are moved to neonatal care immediately after stabilization. If they are born in a local neonatal unit, they are then transferred to a regional neonatal intensive care unit (NICU), where cooling treatment is provided. This may be without their mother if she is not well enough for the transfer. Consequently, parents of these babies experience physical and psychological separation.^3^, ^4^ This is further worsened by the practice of not permitting parents to cuddle their babies until the cooling therapy is completed and the baby is rewarmed to normal body temperature, due to concerns of warming the baby or dislodging breathing tubes or vascular catheters.^5^

Parents have reported that they want to stay with their cooled infants and participate in their care.^4^ Bonding with their babies is also affected when they are not permitted to hold them.^6^, ^7^ NICU nurses identified that parents not holding their babies during cooling added to parents' stress, impacted parent–infant bonding and they would support policies of parents being able to cuddle their babies during cooling therapy.^8^ Early parent–infant separation and uncertainty about long‐term outcome can result in mothers having postnatal depression (around a quarter in one study),^9^ with only half of mothers breastfeeding at discharge and over 80% reporting impaired mother–infant bonding.^10^

Early parent–infant interaction including parental touch,^11^ cuddling or holding is associated with healthy later maternal–infant interactions.^12^ The amount and quality of parental touch influence the psychosocial development in low‐birth‐weight infants.^13^ Therefore, NICUs have embedded family‐centred care enabling parents to have skin–skin contact or cuddling their babies, while receiving intensive care, to promote breastfeeding and bonding and enhance neurodevelopment.^14^

The only published study of ten stable HIE infants treated with therapeutic hypothermia in neonatal care in the United States showed that holding was feasible, resulted in no adverse events and there was positive feedback from mothers and nurses.^15^ We have developed and refined the CoolCuddle intervention that enables parents to cuddle their babies during cooling and intensive care. The CoolCuddle intervention was developed from the cuddling techniques used with parents of babies receiving intensive care and adapted for babies receiving cooling therapy, intensive care and brain monitoring. CoolCuddle was then refined with the first four families in the study by an iterative process of carefully documenting any changes made after each cuddle to produce a standard operating procedure (SOP) involving comments from nurses and parents.^16^ In the CoolCuddle study with 27 cooled babies, cooling temperature, cardiorespiratory and neurophysiology were maintained within clinically acceptable limits.^17^ The aim of this qualitative study was to explore and compare parent and neonatal care staff views and experiences of CoolCuddle in NICU.

## 2 METHODS

### 2.1 Setting

The CoolCuddle study was undertaken in two tertiary NICUs in South West England from October 2019 to December 2020. In 2020, during the COVID‐19 pandemic, we were not able to deliver the CoolCuddle intervention in one NICU due to restricted access. Parent visiting was also severely restricted at this NICU, which prevented both parents from doing any cuddles. Consequently, only six cuddles were delivered in this NICU in 2020, and most of the qualitative data were collected from parents and staff at the other unit.

### 2.2 The CoolCuddle intervention

Parents have their babies on a pillow on their laps, which enables them to interact with the baby and nurses to monitor the baby's well‐being. Before being moved, the wires and tubes around the baby are gathered into two bundles and secured with Velcro at either side of the baby. The baby (with wires) is wrapped in a sheet to keep everything secure, and two (or three) nurses carefully move the baby onto the pillow on the parent's lap. Cuddles can last for up to 2 h. At the end of the cuddle, the baby is moved back to the cot and made comfortable again.^16^, ^17^

### 2.3 Participants

Twenty‐eight families were recruited into the CoolCuddle study, and they undertook over 70 cuddles in total with their babies within the first 4 days; one family was unable to cuddle their baby as the mother tested positive for coronavirus following recruitment and so could not visit her baby during cooling treatment. Infants undergoing therapeutic hypothermia using a servo‐controlled cooling machine and intensive care for HIE were included. Infants were excluded if they needed significant cardiorespiratory support (high‐frequency oscillation, mean airway pressure >15 cm H_2_O, oxygen requirement >70%, in situ chest drain, ≥3 inotropes) or status epilepticus at the time of the planned cuddle.

The first four families involved in refining the intervention were not involved in the qualitative interview study, leaving 23 families as potentially available. Sixteen of these families consented to contact for an interview (70%), but 5/16 families did not respond to any contact about the interview.

Mothers and fathers who had cuddled their baby were invited for an interview by L. B. or J. I. (qualitative researchers) after they had completed their 8‐week outcome questionnaires. Interviews took place online with those who agreed and gave informed consent between March and November 2020. Neonatal staff (nurses and consultants) were also invited by L. B. to take part in interviews from both sites. Interview topic guides for parents and staff were informed by the research literature, team discussions and input from our parent advisors, who were parents with experience of having a baby undergoing therapeutic hypothermia in NICU, but did not have a CoolCuddle intervention. Topic guides are available in the Supporting Information File. Parent interviews took between 16 and 45 min (mean: 32.7 min); two families chose to have joint interviews and the others were individual interviews. Staff interviews ranged from 22 to 51 min (mean: 32 min).

### 2.4 Analysis

Trained qualitative researchers (L. B. and J. I.), with extensive experience of evaluating health care services, conducted the thematic data analysis. Interviews were recorded, transcribed verbatim by a professional transcription service and anonymized. Analysis of the data was an ongoing and iterative process using NVivo 11 software to organize and code the transcripts (QSR International Pty Ltd.) after each interview. Transcripts were initially coded by L. B.; parent and staff interviews were coded separately, and themes were generated.^18^ Coding and candidate themes were discussed and developed with the lead qualitative researcher (J. I.) and the qualitative research group (J. I., L. B., D. O., E. C.) at regular intervals during data collection and analysis to achieve consensus.^18^ Six interview transcripts were also read and coded by J. I. to compare and discuss the coding categories. Parent and staff themes were compared by charting them to highlight common overarching themes between parent and staff data. Interviews continued until data saturation was achieved, in that no new themes were arising from the data. The final themes were presented, discussed and endorsed by the study parent advisors.

The study received Research Ethics Committee approval in August 2019 (19/NI/0143) and Health Research Authority approval in August 2019.

## 3 RESULTS

A total of 21 parents were interviewed from 11 families (11 babies), comprising 11 mothers and 10 fathers. Family demographics are shown in Table 1. All babies were born full‐term, with birth weights similar to the average UK birth weight; all were singletons and four were first babies. Ten staff were interviewed: four neonatal consultants and six nurses (Band 5 junior nurses with several years' experience or Band 6 senior nurses); two were males and eight were females, and two were from the unit where access and cuddles were restricted.

**Table 1:** Family demographics

Demographic	11 Babies	
Infant characteristics		
Baby's sex	3 Females (27%); 8 males (73%)	
Median gestational age	39.4 weeks	Range = 36.6–41 weeks
Median birth weight	3.55 kg	Range = 2.83–3.84 kg
Severe HIE	3 (27.3%)	
Mechanical ventilation	10 (91%)	
Central arterial lines	9 (82%)	
Total cuddles	32 (Mother = 20, father = 12)	Range per baby 2–4
Parent characteristics		
Maternal parity	4 Primiparous, 7 multiparous	
Median maternal age	34.0 years	Range 26–40 years
Median paternal age	34.4 years	Range 28–40 years
Ethnicity	1 non‐White, 10 White	4 English not first language
Marital status	4 Single supported, 7 married	
Delivery type	4 Caesarean sections, 3 assisted vaginal deliveries, 4 normal deliveries	

Abbreviation: HIE, hypoxic–ischaemic encephalopathy.

**Figure 1:**
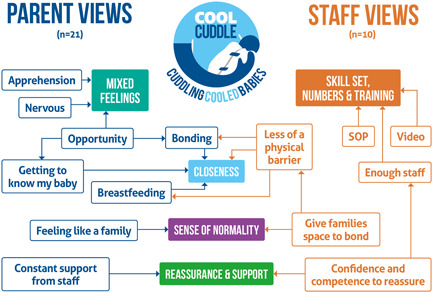
Themes and sub‐themes about CoolCuddle for parents and staff. [Corrections added on 8‐April‐2022, after first online publication: Figures 1 and 2 were interchanged and have been corrected in this version]

All themes are shown in Table 2 and Figure 1. Three themes were comparable between parents and staff: closeness, reassurance and support and a sense of normality. An additional parent theme reflected their mixed feelings about initial participation and a final staff theme discusses the staff skill set, numbers of staff and training needed to undertake CoolCuddle in the NICU environment. Themes are presented with illustrative quotes: Parent quotes are identified by ‘mother or father’ and whether a ‘first baby’; staff quotes are identified by their role.

**Table 2:** Overarching themes comparing parent and staff interviews about CoolCuddle in NICU

CoolCuddle in NICU overarching themes	Parents (*n* = 21)	Staff (*n* = 10)
1.‘Mixed feelings about CoolCuddle’	*An opportunity, apprehension and feeling nervous*	
2. ‘Closeness’	*Getting to know my baby, important for bonding, helped with breastfeeding*	*Less of a physical barrier between parent and newborn; stimulates breast milk production*
3. ‘Sense of normality’	*Feeling like a family*	*Giving families space to bond*
4. ‘Reassurance and support’	*Constant support from staff was valued*	*Conveying confidence and competence to reassure parents*
5. ‘Skill set, staff numbers and training issues’		*Training video, enough staff and clear procedures*

Abbreviation: NICU, neonatal intensive care unit.

**Figure 2:**
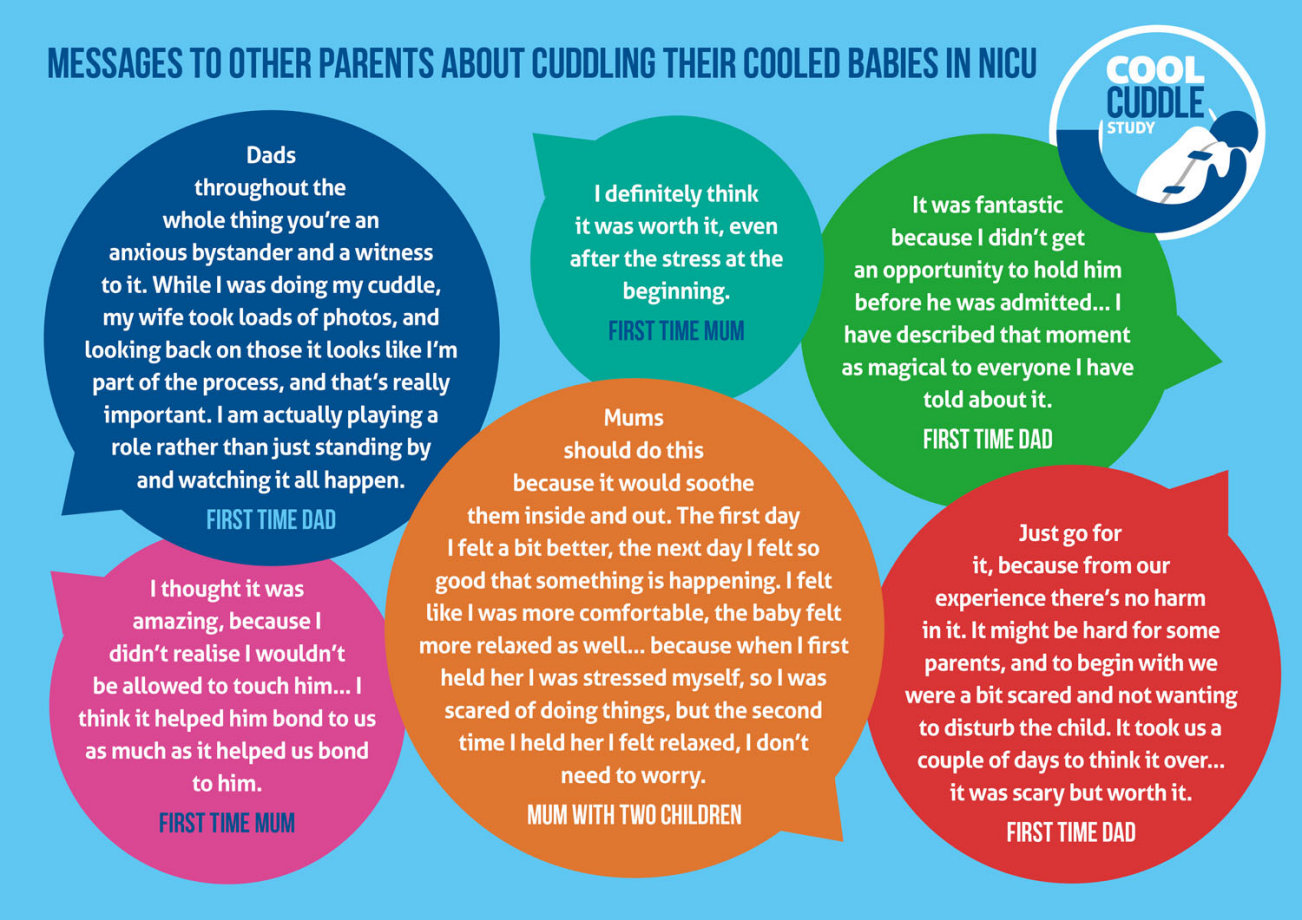
Messages from parents to other parents about cuddling their cooled HIE babies in NICU. HIE, hypoxic–ischaemic encephalopathy; NICU, neonatal intensive care unit

Parents also shared some thoughts to be given to other parents who might be in a similar situation, and these are shown in Figure 2.

### 3.1 Mixed feelings about CoolCuddle

Mothers and fathers described their mixed feelings about being given the chance to hold their cooled baby as overwhelming but also a fantastic opportunity, while initially being apprehensive and nervous of moving their baby from the cot and wondering whether it would be safe with all the tubes and wires connected. Parents' initial anxieties were soon dispelled while holding their baby. Fathers described how they felt this was an opportunity that they had missed out on at the birth and were very grateful to be included in their baby's care. Parents felt reassured that CoolCuddle would not have gone ahead if it was not considered medically safe.

*I think it was a mixture of feelings, hard to describe. It was certainly overwhelming, and it was breath‐taking in some sense, but then also heart‐breaking at the same time, yeah just a mixture of those feelings, how nice it was but at the same time you're closer to reality of what's happening. So it was scary but it was really nice definitely*. (#110, Father)

*It was fantastic because I didn't get an opportunity to hold him before he was admitted, so that was actually the first time I got to hold him, so I was almost as excited about that opportunity as I was about him being born. I was slightly wary about all the equipment… but any anxieties about that disappeared really quickly, and he actually opened his eyes and was staring at me the whole time I was cuddling him, and that was the first time he had consistently opened his eyes while he'd been in hospital*. (#105, Father)

*But that getting closer to what you would expect to be the normal, because I did feel that opportunity to bond had been slightly taken from me by the situation, and so by having the CoolCuddle that gave me some sort of relief that it hadn't been taken away fully*. (#117, Father, 1st baby)

*My main question was just his safety and is it safe to pick up and how long is it safe to hold him for and will the cooling stop and just all of those medical questions, that was what I was most concerned about*. (#110, Mother)

*I was quite concerned with the machines, when they would be ticking and beeping, so that would be a bit of alarm every time something did go off. But then it was just a really proud feeling*. (#121, Father, 1st baby)

*when I said is it safe and stuff, they explained why. (#120, Father)*

### 3.2 Closeness

*Parents* felt that the cuddle gave them a chance to start to get to know their baby, which many had missed out on soon after the birth. Fathers in particular felt that it helped them to bond with their baby and it was important for all of them, mother, father and baby, and the cuddles were described as magical moments. Mothers also felt that the closeness of the cuddle had been important in helping get breastfeeding going.

*We would chat to him and we read books to him and stuff, and then actually it was quite nice to look forward to the cuddle. It was that step closer to hopefully him being better that we were able to hold him, and I think if the worst had happened at least we would have had those cuddles and those pictures*. (#119, Mother, 1st baby)

*It gives me back the bond with my baby. I could even feel his body,.… smell him, kiss him, feel his breath. This is what I actually needed. If I feel like he is uncomfortable and I start talking to him, I start giving a kiss, straight away we can see this cuddling is really good for him*. (#118, Mother)

*I was very grateful for it, and it really helps with all the bonding and everything… an important moment it was my father/son bonding without a doubt… just such a lovely thing after the stressful days that we had, and something I will never forget. I have described that moment as magical to everyone I have told about it*. (#105, Father)

*At one stage he opened his eyes which was really nice, and there were no effects on his heart rate monitor and blood or any of those things, he was still really calm. It was nice, we both bonded really well*. (#110, Father)

*Nurses and consultants* were very positive about the benefits and closeness that cuddling could bring to the parents and baby, helping them overcome the barrier of the cooling jacket with all the wires and tubes and facilitating breast milk production. They felt that it reduced the physical barrier between the parent and the newborn and could ameliorate the negative effects of separation on parental mental health.

*I think there's a significant effect on attachment and maternal and probably paternal mental health from those initial few hours with your baby. Any separation, physical or otherwise is actually very challenging to those relationships*. (Consultant #53)

*I think it really helps improve that bond and removes just the barrier of the jacket. I feel like if they are cuddling them, they don't feel this machinery around them, they are just having a cuddle with their child which is really nice to see….…and that bonding with dad has all the same benefits as the bonding with mum, so it's better for baby and for the parents*. (Nurse #47)

Several *nurses* emphasized the importance of the cuddles in stimulating breast milk production and helped them to encourage mothers to express their milk during the cuddle or soon afterwards. Three *mothers* also talked about breastfeeding and how important it had been to cuddle and be close to their baby to help get breastfeeding going. Some mothers expressed their milk while sitting next to their partners who were holding the baby and others expressed milk soon after their cuddle.

*We didn't have any of the things that we were meant to have done to get good breastfeeding going, and so it felt like having cuddles would mean that I might be able to.… have him closer to me so he can smell me better. I am now exclusively breastfeeding despite all the odds, so I really do think the cuddle has helped*. (#117, Mother, 1st baby)

*When I was trying to get my milk flow going, that massively helped me, just to be close…. I was like I can't get that close to him, so obviously the study really helped, and it definitely did help my milk production come in*. (#105, Mother)

*Before he was discharged from special care, I was able to hold him and start breastfeeding, and actually that [CoolCuddle] probably helped with the transition of not being able to hold him at all to suddenly having him in my arms for a long time and breastfeeding etc, that probably helped with getting used to that really*. (#121, Mother, 1st baby)

*Once they have had a cuddle we always say to Mum go and express, it's a really good time to express, that closeness and that bonding and being able to see and smell their baby is really good for the milk production and things*. (Nurse #63)

*It's such a wonderful moment the first cuddle, we get lots of lovely photos, and then each of the parents or the mummy in particular to look at those when she's expressing her milk to feel the closeness of her baby*. (Nurse #55)

### 3.3 Sense of normality

*Parents* described having the cuddles as helping them to feel a bit more normal, to start to feel like a family and to share their baby with their wider family. They could also start to have more normal conversations with their family and friends about their baby while having a cuddle.

*That's probably the first time we started talking about what it feels like to be a new parent, we even almost tried to move to a normal conversation rather than just talk about what's wrong and what happened, almost normalising the situation. We would use that time to call family and do a Facetime video and I think that was really important for us and for the family as well*… (#121, Mother, 1st baby)

*So even though I knew [partner] had one [cuddle] the day before, but it was just like yeah finally felt it was a bit of normality there*. (#119, Mother, 1st baby)

All the *staff* mentioned the importance of close contact for parents to bond with their baby and, if possible, to leave them to have some quiet family time during the cuddle to start the process of getting to know their baby while still being able to monitor the baby.

*I would sit and stay with parents for at least the first 20 minutes that the baby is out, just check that they are really settled and they are not getting uncomfortable, that the parents were settled, and chat, and then I would probably step back a bit and leave them to have a bit of bonding time, because you never get time with your own child on your own in NICU otherwise, a nurse is always lurking and you don't really get to bond*. (Nurse #50)

### 3.4 Reassurance and support

*Parents* valued having the NICU nurses and the research team around all the time while they were cuddling their baby. This constant calm staff presence was very reassuring for them to explain what was happening when alarms went off and check that all was well. Continued reassurance and support dissipated some of the negative emotions felt at the beginning of the cuddle and enabled parents to bond and establish a sense of some normality.

*The whole thing it was just really reassuring to be able to get him out and it was positive that he was okay coming out of that incubator. Yeah it was all just very reassuring and nice*. (#120, Mother)

*Really lovely reassurance, and people checking that we did understand, …I felt very reassured and confident in what they were doing. I think it's important to have somebody who has got a really calm and patient way to communicate with the parents*. (#105, Mother)

*I think for me it's just that constant reassurance is helpful*… *just little updates on his stats are good, or his brain activity is looking normal, just so you know that it's not doing anything different whilst you're holding him*. (#109, Mother)

*Staff* also mentioned that it was important to support parents in a confident way when they were cuddling their babies. They felt that parents needed a lot of reassurance and discussion around the cuddle being done in a controlled, safe way with the nursing team around all the time, and if there were any concerns at any point then they would stop. Conveying this confident approach helped parents feel that it was a positive experience for them.

*I think just really good communication, what the process is going to be in terms of what we want them to do, where we want them to sit, if we're going to put a pillow underneath them or not, just making sure they are comfortable. Making it clear to them where they can touch and what parts of their baby they can touch, because I think sometimes parents can feel a bit afraid to touch their babies…. a lot of time that's just reassurance*. (Nurse #63)

*So there was a lot of reassurance that was involved I would say, which you can understand. But I would say there was also a lot of enthusiasm for it, and when I spoke to parents after it all they all felt it was quite a positive experience. I think it was just the reassurance of how it was going to happen that took the most time I would say*. (Consultant #74)

### 3.5 Skill set, staff numbers and training issues

All the staff mentioned the importance of having enough staff available and good training in moving cooled babies, so that they could conduct the moves safely. These were their greatest concerns and so encouraged the idea of a video showing the whole process that could be used for teaching the nursing staff, in addition to hands‐on simulated or practical training opportunities led by experienced nursing staff. This would enable skills and expertize to be passed on to other members of the nursing team. Consultants felt that this was primarily a nursing intervention so the training should be for them and endorsed the intervention as enhancing family‐centred care.

*It's important for staff to know which babies are suitable, how to explain things to parents, what parents need to know, and the best way of making sure everything is secure. So making sure there's education, some videos of how to do it and taking pictures……I think the main things is staffing, just making sure you've got three members of staff that are free to get the babies out and to also sit with parents and be with parents while they are having their cuddle and reassuring them*. (Nurse #63)

*I definitely think the barriers will come with staffing and whether people have the time and the skill mix to be able to move them…. You need extra people to move them, but once they are in a settled position for the cuddle you can go back just to the original nurse that was looking after them. I think you would still need someone a bit more experienced and who is comfortable with moving these babies to troubleshoot with them. But I do think a video is quite a good way of practically showing this is how we should do it with a SOP and checklist*. (Nurse #47)

*I think it's about feeling there's a fully integrated plan between the nursing team and the parents, so the parents have confidence. I think parents with babies coming out for cuddles, whether that's the preterm babies or the cooling babies, are anxious of having a baby who is attached to a load of stuff, and what will happen if we dislodge [it]. If you can set out clearly what's going to happen you can do it in a controlled way with the right staff and the right number of staff involved, and it's nicely protocolised, I think that's what parents need*. (Consultant #67)

## 4 DISCUSSION

This study has shown the importance to parents of cuddling their sick cooled babies while they were undergoing therapeutic hypothermia. Despite being apprehensive about taking part, parents found it to be a wonderful opportunity and enjoyed the closeness, which helped them get to know and bond with their baby that often felt taken away from them at birth. Some mothers felt that this closeness also helped stimulate breastfeeding. Parents felt that the cuddles helped them start to feel a bit more normal and move on as a family and valued the reassurance and support from the nursing staff at this time. Staff felt that CoolCuddle helped parents to have less of a physical barrier between them and their baby and echoed the consequences for breastfeeding. They felt that it was important to be able to convey confidence and competence to reassure parents, giving families the space to bond, and to do this would need adequate staff numbers, good training materials including a video, practical training opportunities with experienced members of staff and a detailed SOP. Having a number of nursing staff trained in CoolCuddle procedures was important, to maximize opportunities for parents to cuddle their baby. To this end, we have developed a video showing the entire process of the CoolCuddle and a detailed SOP with pictures of moving cooled HIE babies and parents cuddling them, to support the explanation in the SOP. We also have a clear checklist that was developed and refined during the study.

Other studies exploring parents' perceptions of therapeutic hypothermia for HIE confirm parents' concerns about bonding following their traumatic experiences and wanting more active involvement with their infant, more information and emotional support from staff.^4^, ^7^, ^8^, ^10^ Delivering therapeutic hypothermia in the context of family‐centred care practices in the NICU will help parents' transition to parenthood by involving them in nursing care and decisions.^19^ Enabling CoolCuddle to take place during therapeutic hypothermia will help to strengthen and enable family‐centred care practices for these infants in the NICU. Facilitating such periods of cuddling cooled infants will facilitate parent–infant bonding, breastfeeding and perhaps reduce levels of postnatal depression amongst mothers. Fathers in our study were very positive about having the opportunity to support their partner and take on an active role in their baby's care at a very difficult time, when many had felt like a bystander up until that point. They emphasized that it is important to remember that fathers need to bond with their babies at this traumatic time for the family. Addressing the staffing challenges should be supported by good training (including the video and SOP) and support from existing staff with experience of CoolCuddle.

### 4.1 Study strengths and limitations

The strengths of this study include conducting interviews with over half of the parents involved in CoolCuddle and gaining experiences from both mothers and fathers. This is the first study to allow fathers to hold their cooled HIE infant and to capture their views of the experience. Another strength was having parent advisors involved throughout the study to guide the interviews and discuss the findings. Limitations include the restrictions due to the COVID‐19 pandemic, which precluded face‐to‐face interviews that might have provided more rich discussions, and the fact that fewer nurses took part in interviews than we had hoped, which limited staff views.

## 5 CONCLUSIONS

Parents being able to cuddle their baby during cooling treatment for HIE in NICU was positively received by parents and neonatal staff as helping to facilitate parent–infant bonding and family‐centred care. Fathers particularly appreciated the opportunity to hold and bond with their baby. Adverse perinatal mental health, impaired mother–infant bonding and their effects on the establishment of breastfeeding may be ameliorated by introducing CoolCuddle, following a period of careful evaluation in more NICUs.

## CONFLICTS OF INTEREST

The authors declare that there are no conflicts of interest.

## Supporting information

Supporting information.

Supporting information.

## Data Availability

The data that support the findings of this study are available from the corresponding author upon reasonable request.
