# Supplementary material for: ‘Opportunity to bond and a sense of normality’: Parent and staff views of cuddling babies undergoing therapeutic hypothermia in neonatal intensive care: ‘CoolCuddle’
Source: Health Expect. 2022 Mar 24;25(4):1384–92. doi: 10.1111/hex.13477 (PMC9327856; doi:10.1111/hex.13477)
Supplement: Supplementary file 1 — Supporting information. [file HEX-25--s002.doc]

Parents will be contacted by telephone. Confirmation of consent to take part and for interview to be recorded – this will be recorded verbally. Topics to be covered:

1. **Experiences of their stay in NICU:**
   1. Was this a local hospital (Bristol) or did you come from another town?
   2. How long did your baby stay in NICU?
   3. Do you have any other children and any experience of NICU?
   4. What did you think about the CoolCuddle study when you were first told about it?
   5. How did you feel about holding [name] while they were cooling?
   6. How do you think the process has helped you to bond with [name]?
   7. What [if anything] reassured you [*Before, During] the cuddle?*
   8. What was explained to you by the staff? *Before, During, After cooling*
   9. Were you comfortable?
   10. Do you think [name] was comfortable, content?

[DADS: explore if CoolCuddle important for dad if they didn’t get to hold baby when born]

1. How many times did you hold [name] while they were being cooled?
2. Did you hold [name] while they were being warmed up after cooling?
3. Have you and [partner] discussed how you felt about holding [name] while they were being cooled? *[joint reflections on holding baby]*
4. **If [partner] not being interviewed:**
5. How did [partner] feel about holding [name] while they were cooling?
6. How many times did [partner] hold [name] while they were being cooled?
7. Do you think the process has helped [partner] to bond with [name]?
8. **Now you are at home:**
   1. What impact has holding your baby had on what you do and how you feel?
   2. When is the best time to approach parents about the CoolCuddle study?

[was their approach well-timed?]

- 1. **Is there anything that you would like other parents to know about the process?**

Is there anything else it is important for us to know (about holding a cooled baby) that might help staff make the process better for parents?

This is a guide only. Interviewer to use further prompts and probes in response to parents’ responses. Interviewer to use own discretion to omit questions or alter wording as appropriate during interviews.
