# Supplementary material for: ‘Opportunity to bond and a sense of normality’: Parent and staff views of cuddling babies undergoing therapeutic hypothermia in neonatal intensive care: ‘CoolCuddle’
Source: Health Expect. 2022 Mar 24;25(4):1384–92. doi: 10.1111/hex.13477 (PMC9327856; doi:10.1111/hex.13477)
Supplement: Supplementary file 2 — Supporting information. [file HEX-25--s001.doc]

1. **How long have you worked in NICU?**

[Yrs experience in current NICU & other locations / any prior experience of CoolCuddle?]

**Staff experience of CoolCuddle**

1. **How many CoolCuddle families were you involved with in total?**
2. **Can you describe your experience of [talking with parents/moving] that particular baby?**

[clarify who was involved, who did what - extent of involvement: observing, talking to parents/other staff about the process, holding equipment/wires when research nurse (RN) moved baby, or actually moving baby with assistance from RN/other staff]

1. **How did you find the process [of moving the baby]?**

[level of involvement - acceptable/difficult, level of input needed from other nurses/consultants]

1. **What is different about CoolCuddle? (to day-to-day moving of babies)**

[Equipment wise, if not discussed any similarities to moving non-cooling NICU babies?]

1. **How might CoolCuddle ‘fit’ in NICU on day-to-day basis?**

[Level of staff input required]

1. **Any concerns you have about CoolCuddle?**

[Own views about baby staying still in incubator versus moved to parent’s arms/stimulation, safety?]

**Parents experience of CoolCuddle**

1. **What do you think parents need to know about CoolCuddle?**
2. **What did you explain to parents about moving the baby?**

[***Before, During, After cooling***, if they were not involved in these explanations would they feel able to explain aspects of CoolCuddle to families?]

1. **What questions did parents have about their baby being moved/cuddled?**

[***Before, During, After cooling***, who answered these questions if they were not involved e.g. research nurse, consultant neonatologist etc?]

1. **How do you think CoolCuddle might have helped baby?**
2. **How do you think CoolCuddle might have helped parents?**

[clarify both mum and dad]

1. **What could be done to improve the CoolCuddle process for parents?**

**Staff training**

1. **What training did you have in relation to CoolCuddle?**

[before/during (what to look out for/ what happens after) process, was this enough?]

1. **What training would you have liked?**
2. **What training do you think other staff will need?**
3. **We are in the process of developing video….**

[their views on a training video for: CoolCuddle families /and staff, step-by-step process of moving baby]

1. **What would you need to make the CoolCuddle process better for staff?**
2. **Is there anything else it is important for us to know?**
